# Granulomatous inflammation mimicking a hematoma around the replaced ascending aorta in magnetic resonance imaging: a case report

**DOI:** 10.1186/s13019-023-02298-y

**Published:** 2023-06-13

**Authors:** June Lee, Hyun Ah Lim, Seok Beom Hong, Do Yeon Kim, Yong Han Kim, Hwan Wook Kim

**Affiliations:** Department of Thoracic and Cardiovascular Surgery, Seoul St. Mary’s Hospital, College of Medicine, The Catholic University of Korea, 222 Banpo-daero, Seocho-gu, Seoul, 06591 Republic of Korea

**Keywords:** Granulomatous inflammation, Magnetic resonance imaging, Thoracic aortic surgery

## Abstract

**Background:**

Granulomatous inflammation results from various causes including infections and allergic reactions. It can appear as high signal intensity in T2-weighted or contrast-enhanced T1-weighted magnetic resonance imaging (MRI). Here, we describe a case of granulomatous inflammation looking like a hematoma on an ascending aortic graft in MRI.

**Case presentation:**

A 75-year-old female was undergoing assessment for chest pain. She had a history of hemi-arch replacement for aortic dissection 10 years earlier. The initial chest computed tomography and subsequent chest MRI were suggestive of a hematoma, implying a pseudoaneurysm of the thoracic aorta, which is associated with high mortality in reoperation. Through redo median sternotomy, severe adhesion was found in the retrosternal space. A sac in the pericardial space contained yellowish and pus-like material, confirming that there was no hematoma around the ascending aortic graft. The pathologic finding was chronic necrotizing granulomatous inflammation. Microbiological tests including polymerase chain reaction analysis were negative.

**Conclusion:**

Our experience indicates that an MRI finding of a hematoma at the site long after cardiovascular surgery suggests that there may be granulomatous inflammation.

## Background

As a specific form of chronic inflammation, granulomatous inflammation can be caused by infections, allergic reactions, and neoplastic conditions. It presents with mononuclear leukocytes, specifically macrophages, responding to cell injury [1]. This histologic response especially affects the lungs, skin, kidneys, liver, and lymph nodes, although it can occur in all tissues [2].

On magnetic resonance imaging (MRI), T2-weighted or contrast-enhanced T1-weighted high signal intensity can be found in granulomatous inflammation [3]. T1 or T2 mapping may be useful for recognizing granulomatous tissue infiltration of cardiac tissue, like sarcoidosis [4].

Herein, we illustrate a rare case of granulomatous inflammation on a replaced ascending aorta, which showed as a perigraft hematoma in MRI.

## Case presentation

Ten years postoperatively, a 75-year-old female presented to the emergency department with a history of seven days of chest pain without fever. She had a background of hemi-arch replacement with a Hemashield platinum woven double velour vascular graft (collagen-impregnated polyester graft, Getinge AB, Lindholmspiren 7 A, Göteborg, Sweden) for ascending aortic dissection 10 years earlier. There was no history of prior trauma.

**Fig. 1:**
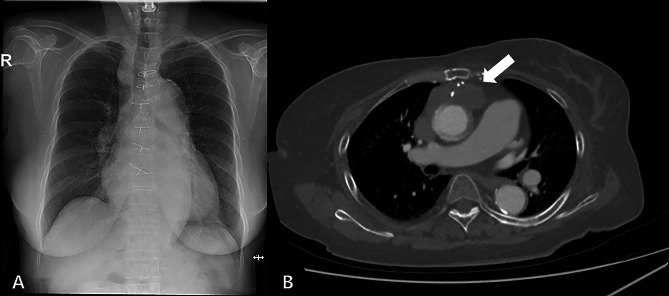
The initial findings on imaging studies. (**a**) Cardiomegaly was detected on the chest x-ray. (**b**) Initial chest computed tomography showing a mediastinal mass, possibly a hematoma (white arrow)

The chest x-ray showed evidence of cardiomegaly (Fig. 1A), and the initial chest computed tomography (CT) was suggestive of hematoma or abscess (Fig. 1B). The size of the mediastinal mass was about 4.5 × 3.3 cm. In patients with previous aortic surgery, the existence of a radiological perigraft hematoma means clinically that there can be an ascending aortic pseudoaneurysm, which is an uncommon complication of cardiac surgery but could be catastrophic in 0.5% of the patients [5]. And it can occur in a variety of locations including previous anastomotic sites, and cannulation and venting sites [6].

**Fig. 2:**
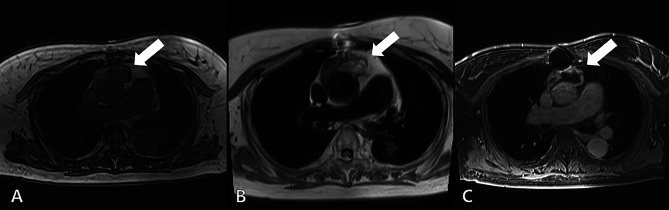
Preoperative chest MRI. (**a**) T1-weighted image suggestive of hematoma (white arrow). (**b**) HASTE T2-weighted sequence images suggesting the possibility of a pseudoaneurysm (white arrow). (**c**) T1 contrast image with rim enhancement (white arrow) MRI, magnetic resonance imaging; HASTE, half-Fourier single-shot turbo spin-echo

Despite the necessity of promptly diagnosing a pseudoaneurysm of the thoracic aorta and carrying out surgical intervention, we proceeded with caution during our preoperative assessment. Chest MRI was deemed essential to facilitate the diagnosis. The MRI scan with enhancement revealed a hematoma on the ascending aortic graft consistent with the CT findings (Fig. 2A and B). An infection could not be excluded because there was rim enhancement suspicious of an abscess on the contrast image (Fig. 2C). The blood culture of the patient was negative and other diseases causing chest pain were also ruled out.

**Fig. 3:**
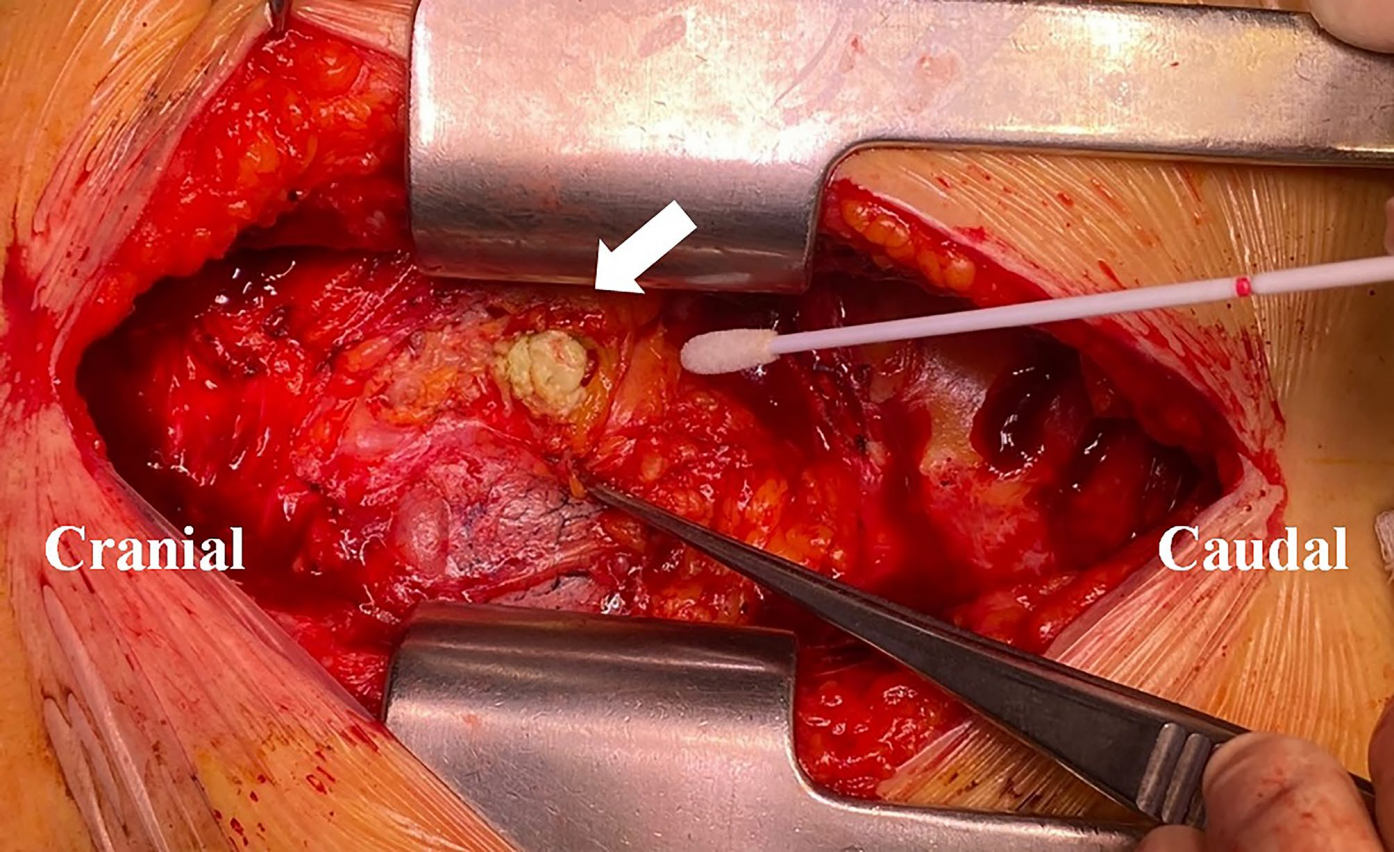
Intraoperative findings revealing yellowish material in the pericardium (white arrow)

A well-prepared surgery was planned in case of a need for redo aortic surgery. After endotracheal general anesthesia, a repeat median sternotomy was performed, revealing severe adhesion in the retrosternal space. Inspection of the pericardial space showed a sac containing yellowish and pus-like material (Fig. 3). We confirmed that there was no hematoma around the ascending aortic graft. After collecting the material for culture and biopsy, the sac was removed and massive irrigation was performed. The chest wall was closed after chest tube insertion.

**Fig. 4:**
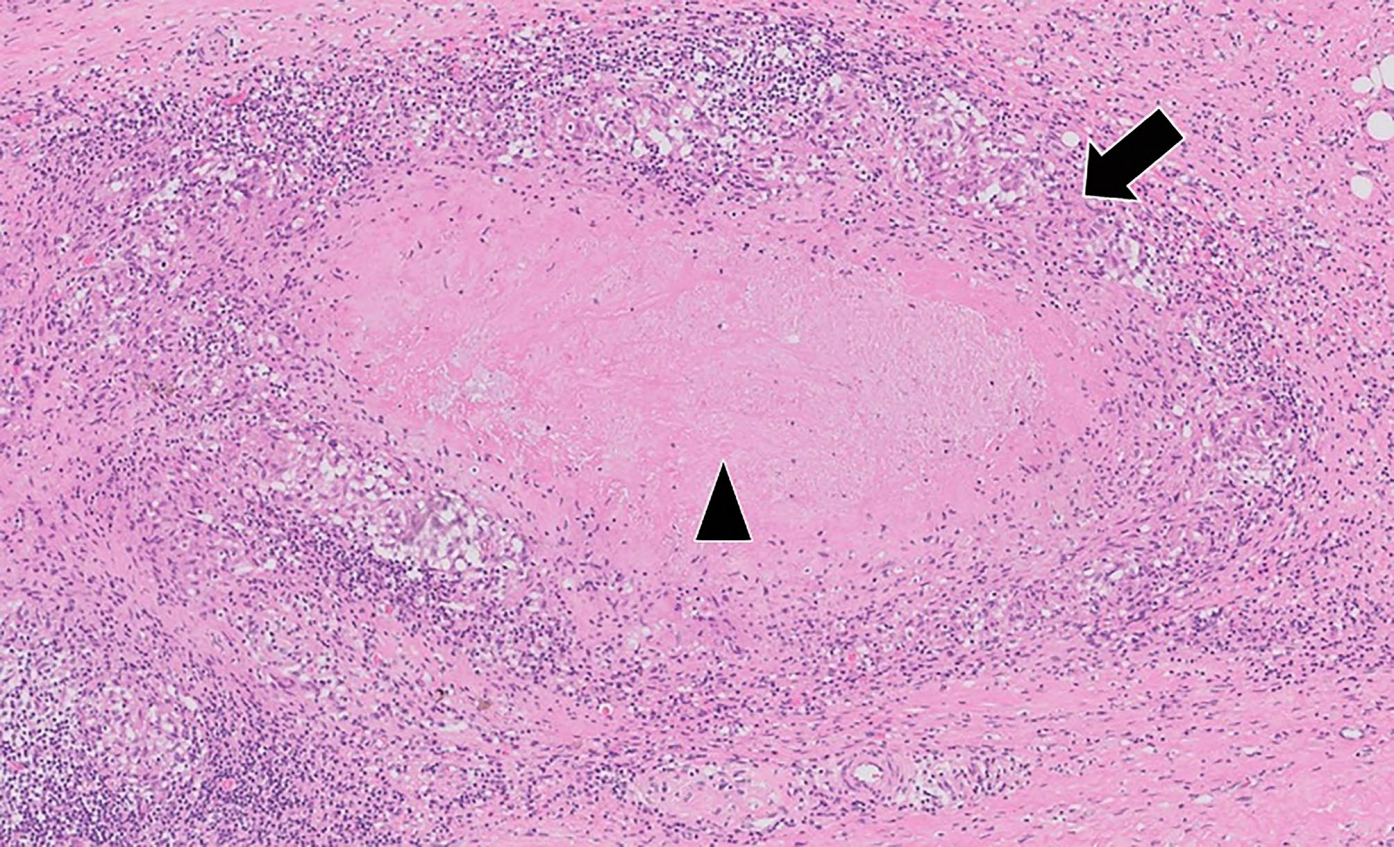
The pathologic findings (H&E stain) demonstrated granulomatous inflammation with a rim of histiocytes (black arrow) and central necrosis (black arrowhead) H&E, hematoxylin and eosin

The pathologic findings showed chronic granulomatous inflammation with necrosis (Fig. 4). The microbiologic tests of the tissue specimen including cultures and polymerase chain reaction analysis for *Mycobacteria tuberculosis* complex and non-tuberculosis *Mycobacteria* were negative.

The patient’s postoperative course was uneventful, and he was discharged on postoperative day 7 after confirmation by the infection specialist. No symptom was noted at the outpatient department follow-up.

## Discussion

We encountered a case where the preoperative differential diagnosis was very important for surgical planning. In the imaging findings of patients who underwent previous cardiovascular surgery, a radiological hematoma on the anastomosis sites may mean a pseudoaneurysm clinically. Pseudoaneurysm, with the disruption of at least one layer of the wall of the vessel around the thoracic aorta [7], has a variety of causes, including trauma, infection, and suture dehiscence [8], and that would lead to a risky reoperation frequently [9]. Multiple studies have reported that redo procedures involving the aortic root and proximal aorta were associated with high mortality rates [10–12].

Even though CT angiography is most commonly used to assess the aorta as the modality of first choice [13], cardiovascular magnetic resonance imaging (CMR) was specifically recommended in American Heart Association/American College of Cardiology guidelines and can play a vital role in many clinical scenarios [14]. It is also recommended for the identification of acute aortic disease conditions, as well as the monitoring of stable and moderate states [15]. CMR can help to estimate the age of a vessel wall hematoma and review signal intensities, which vary over time [16].

In our case, the granulomatous inflammatory subtype pattern was necrotizing granuloma. On MRI, chronic granulomatous inflammation can be found as ring-enhancing lesions [3], as in our case (Fig. 2C). Foreign body-like synthetic fibers can also cause granulomatous inflammation [2].

## Conclusion

Our experience showed that an MRI finding of a hematoma at the site long after cardiovascular surgery suggests that there may be granulomatous inflammation. It is hoped that this case will help surgical planning in similar cases.

## Data Availability

As this paper is a case report, all generated or analyzed data are included in this article.

## Abbreviations

MRI: Magnetic resonance imaging
CT: Computed tomography
CMR: Cardiovascular magnetic resonance imaging.
